# Supplementary figures and images for: PLANES: Plausibility analysis of epidemiological signals
Source: PLoS One. 2025 Mar 28;20(3):e0320442. doi: 10.1371/journal.pone.0320442 (PMC11952232; doi:10.1371/journal.pone.0320442)

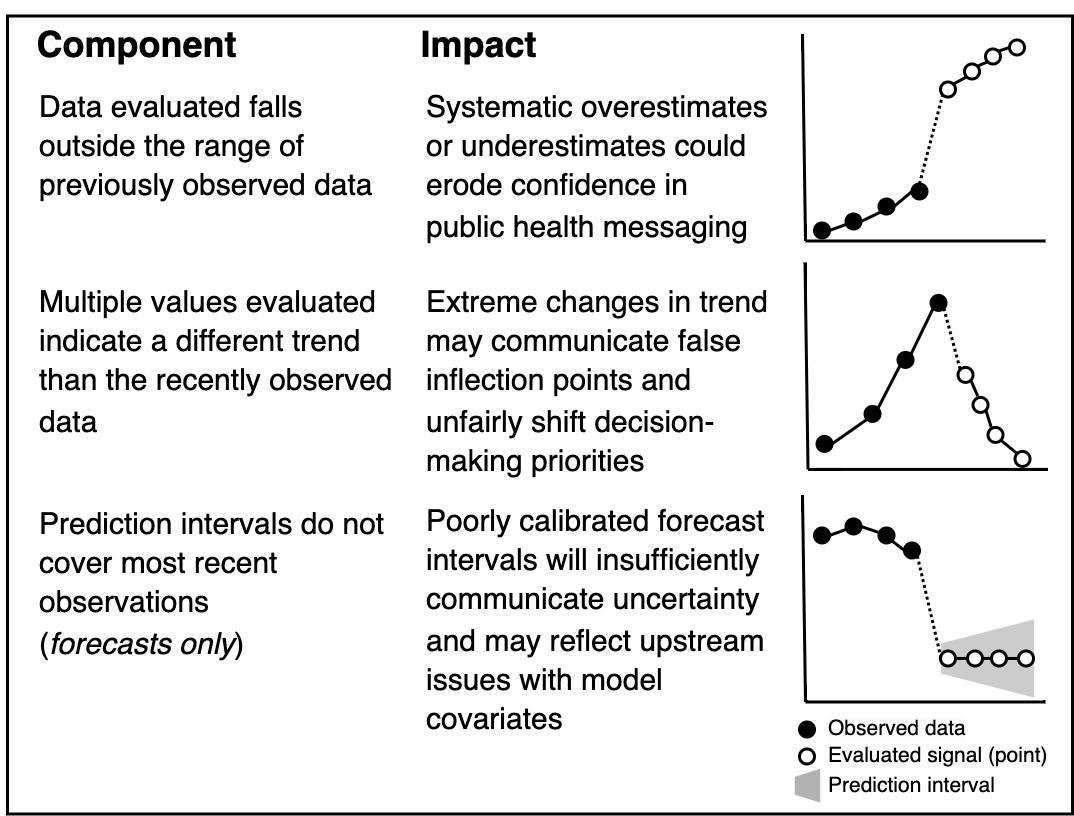

Supplement: S1 Fig — Illustration of the conceptual motivation for developing the PLANES approach. Several examples of possible components and their impacts are described. (TIFF) [file pone.0320442.s001.tiff]

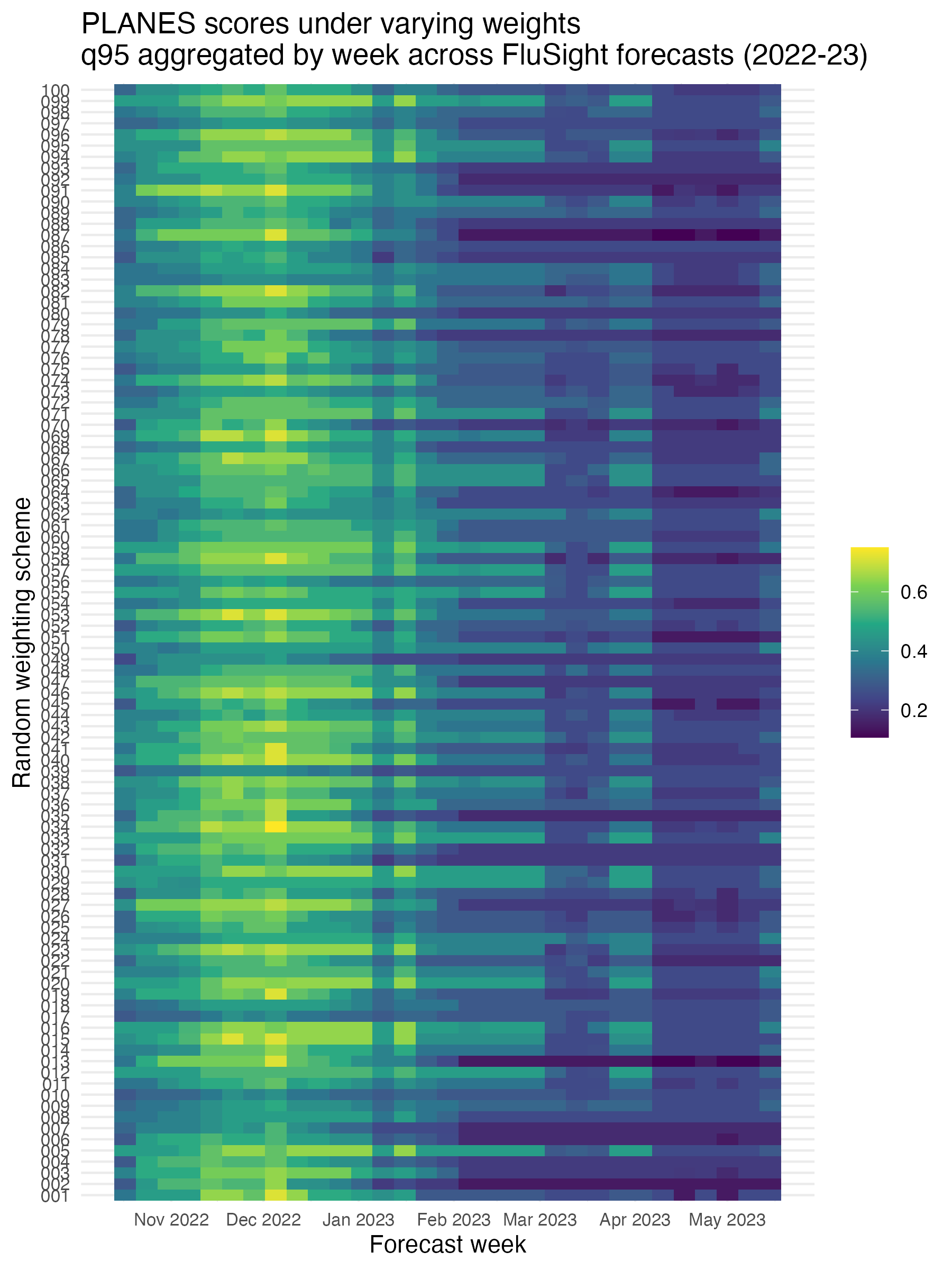

Supplement: S2 Fig — Demonstration of the sensitivity of PLANES scoring to varying weighting schemes. All eligible FluSight forecast submissions in the 2022-23 season were scored using PLANES under 100 randomly sampled weighting schemes. For each forecast week, the scores were aggregated across all forecasters and locations and summarized to identify the 95th percentile (q95). The tile plot is shaded by q95 at each week under the 100 different weighting schemes. At certain points in the season scores are consistently higher or lower across weighting schemes. For example, PLANES scores for late season forecasts have a lower q95. However, the weighting schemes visibly shift the q95 threshold for many forecast weeks. (TIFF) [file pone.0320442.s002.tiff]

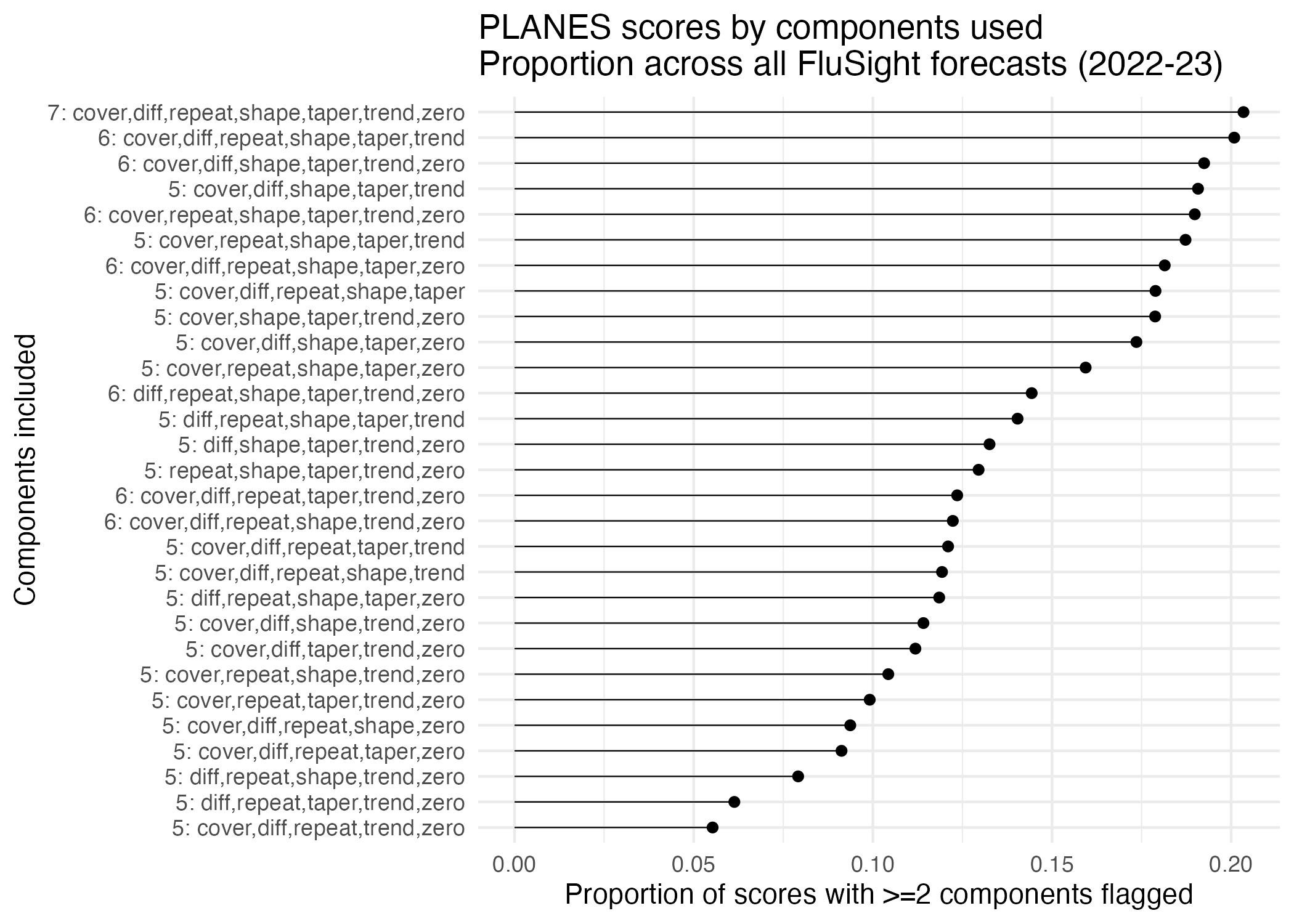

Supplement: S3 Fig — Impact of PLANES component sets on overall scoring. All eligible FluSight forecast submissions in the 2022-23 season were scored using all combinations of PLANES components. Across all forecast weeks, forecasters, and locations, we counted the number of PLANES scores with at least two flags raised using each component set. In this figure, the results are restricted to component sets with between five and seven components. Within sets that had the same overall number of possible components, there was a wide range in the proportion of scores that had at least two flags raised. For example, the proportion ranged between 0.06 and 0.19 among sets with five components. In some cases, sets with fewer components had a higher proportion. (TIFF) [file pone.0320442.s003.tiff]
