# Supplementary material for: PLANES: Plausibility analysis of epidemiological signals
Source: PLoS One. 2025 Mar 28;20(3):e0320442. doi: 10.1371/journal.pone.0320442 (PMC11952232; doi:10.1371/journal.pone.0320442)
Supplement: S1 File — Equations and examples for each of the seven PLANES components. (PDF) [file pone.0320442.s005.pdf]

Additional details on PLANES components

Difference component

The formula in Equation 1 demonstrates how the maximum difference (μ) is computed, with X as the observed signal used to create the seed, t being the time step, and i representing the the number of steps in the time series.

$$\mu = max(|X_{t=1} - X_{t=0}|, |X_{t=2} - X_{t=1}|, ..., |X_{t=i} - X_{t=i-1}|) \tag{1}$$

For each horizon (h) in the time steps (j) for the evaluated signal (Y), μ is compared to the computed difference between h and $h-1$. As we specify in Equation 2, the algorithm checks if any of the differences exceed μ.

$$any(|Y_{h=1} - X_{t=i}| > \mu, |Y_{h=2} - Y_{h=1}| > \mu, ..., |Y_{h=j} - Y_{h=j-1}| > \mu) \tag{2}$$

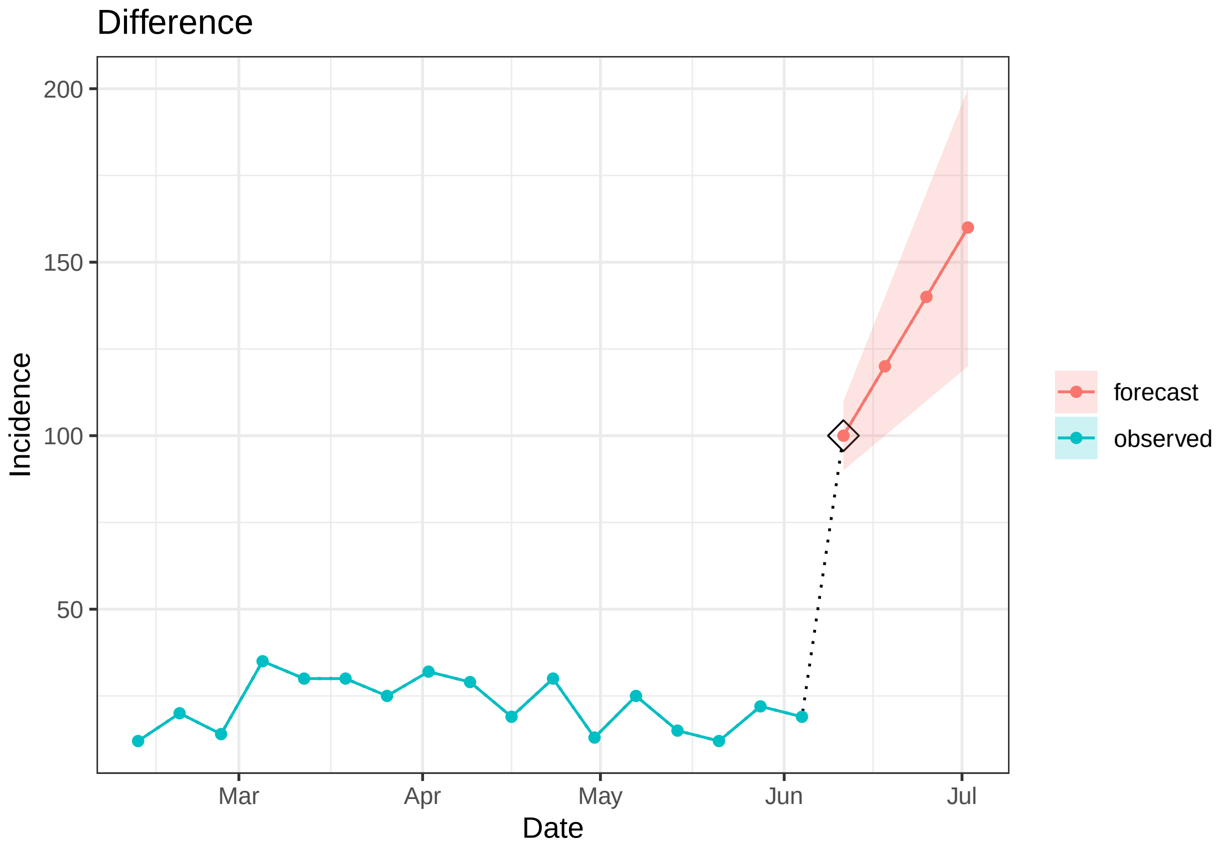

Depiction of a flag raised with the difference component. The difference component checks the point-to-point differences for evaluated signal. This component can be used on either forecasts or observed signals. The function internally computes the maximum observed difference (using absolute value) and checks to see if any of the point-to-point differences for the evaluated data exceed that threshold.

Coverage component

Equation 3 specifies the formula for the coverage component, with the minimum and maximum of the forecasted prediction interval (ϕ) at the first horizon assessed against the last observation of the observed signal.

$$X_{t=i} < min(\phi_{h=1}) \lor X_{t=i} > max(\phi_{h=1}) \tag{3}$$

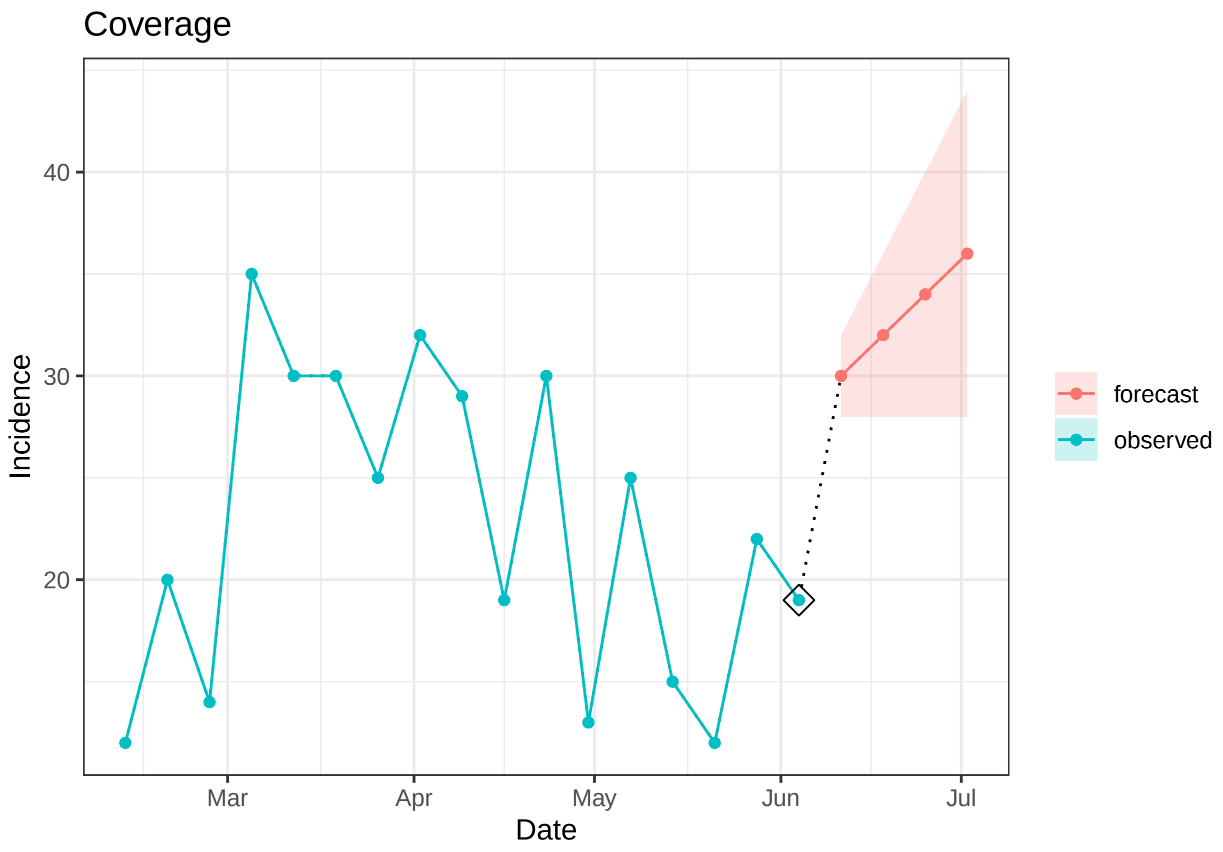

Depiction of a flag raised with the coverage component. The coverage component compares the prediction interval for the first horizon of the evaluated signal to the most recent value in the seed. If the interval does not cover the most recent data point, then the flag is raised as implausible. Because this component requires a prediction interval, it can only be used to assess plausibility of forecast signals.

Taper component

Equation 4 describes the algorithm for comparing the width of prediction interval (ϕ) at each horizon (h) to the corresponding width at every consecutive time step (j).

$$any(max(\phi_{h=2}) - min(\phi_{h=2}) < max(\phi_{h=1}) - min(\phi_{h=1}), ..., max(\phi_{h=j}) - min(\phi_{h=j}) < max(\phi_{h=j-1}) - min(\phi_{h=j-1})) \quad (4)$$

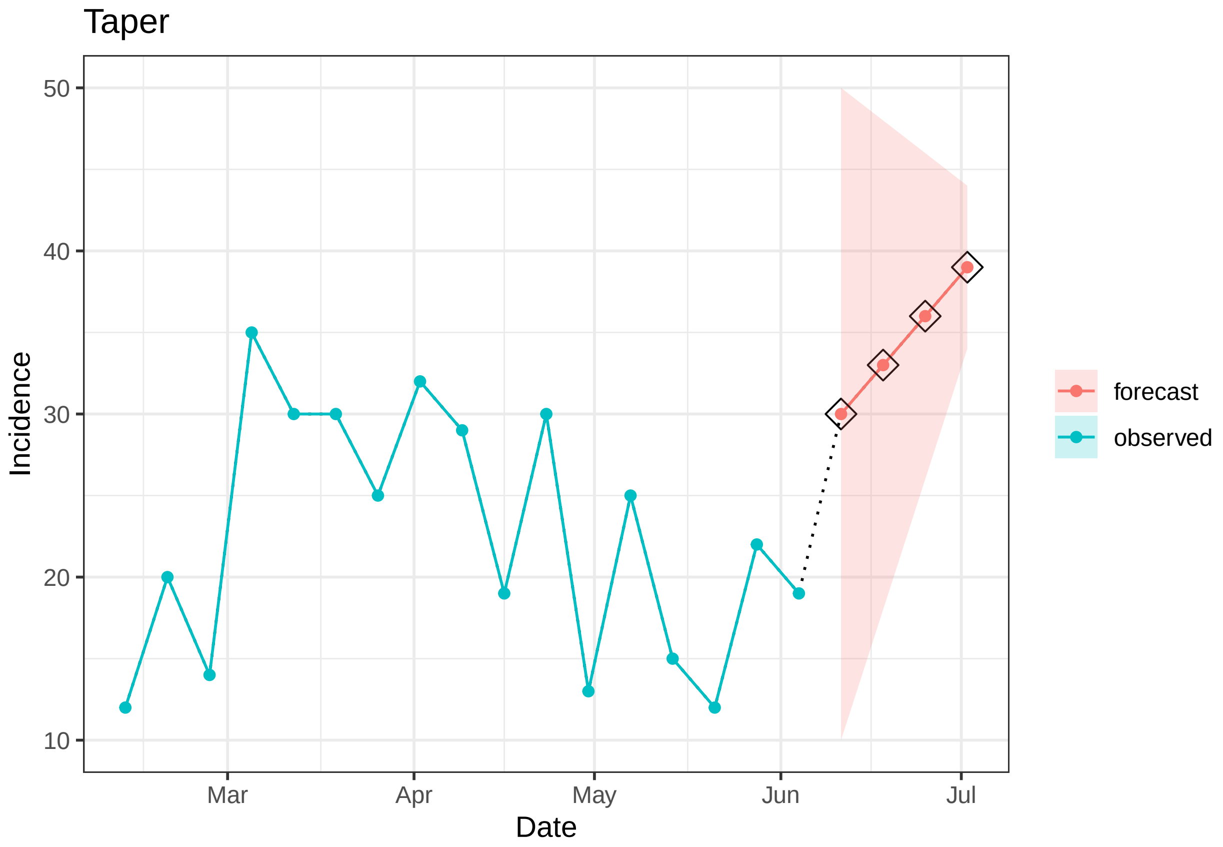

Depiction of a flag raised with the taper component. The taper component checks whether the prediction interval for the evaluated signal decreases in width (i.e., certainty increases) as horizons progress. Because this component requires a prediction interval, it can only be used to assess plausibility of forecast signals.

Repeat component

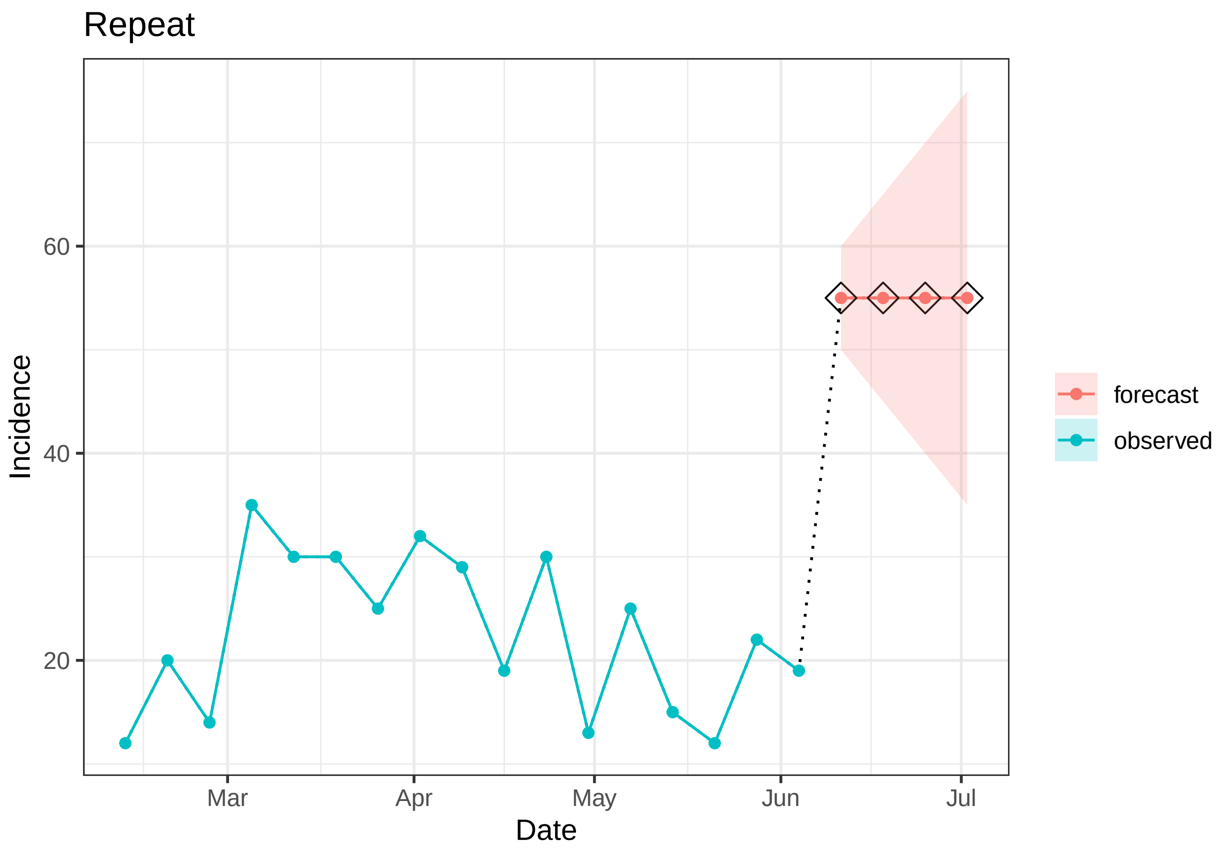

Depiction of a flag raised with the repeat component. The repeat component checks whether consecutive values in an observed or forecasted signal are repeated k times. When the seed is created, it stores the maximum number of consecutive repeats for each location and uses this as the default value for k. If the evaluated data exceeds k then the signal is considered implausible and a flag is raised.

Trend component

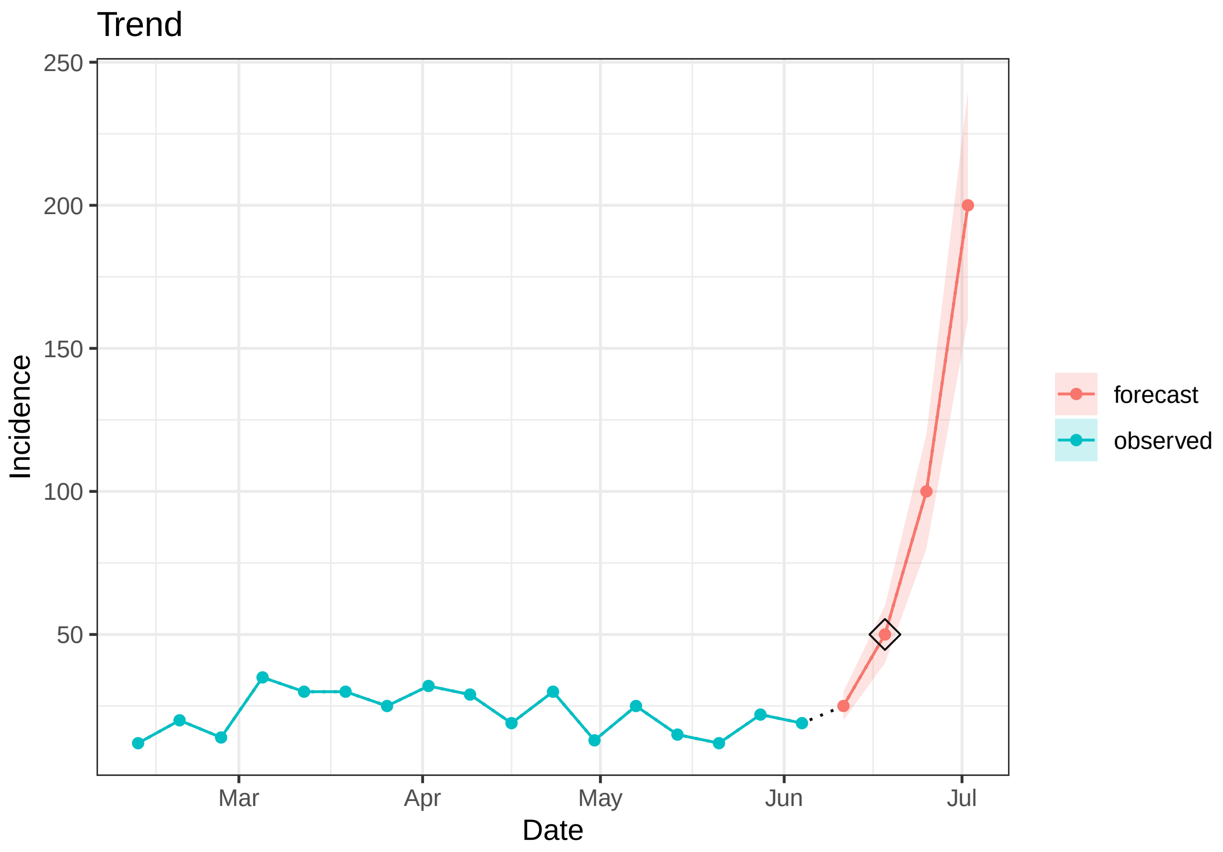

Depiction of a flag raised with the trend component. The trend component assesses whether there is a significant change in the magnitude or direction of the slope for the evaluated signal compared to the most recent data in the seed. If a "change point" is identified in any of the forecasted horizons and/or the most recent seed value, then the flag is raised for implausibility.

Shape component

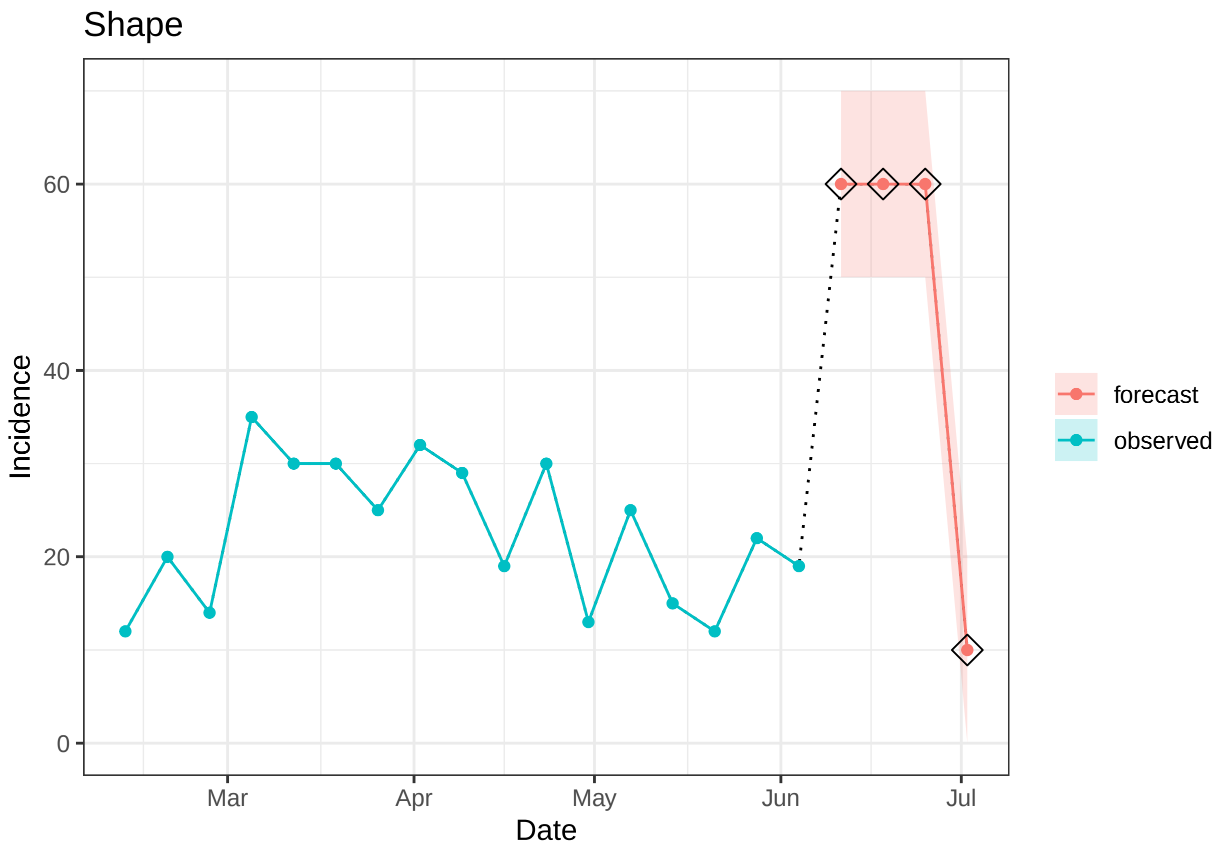

Depiction of a flag raised with the shape component. The shape component evaluates the shape of the trajectory of the forecast signal and compares that shape to existing shapes in the observed seed data. If the shape is identified as novel, a flag is raised, and the signal is considered implausible.

Zero component

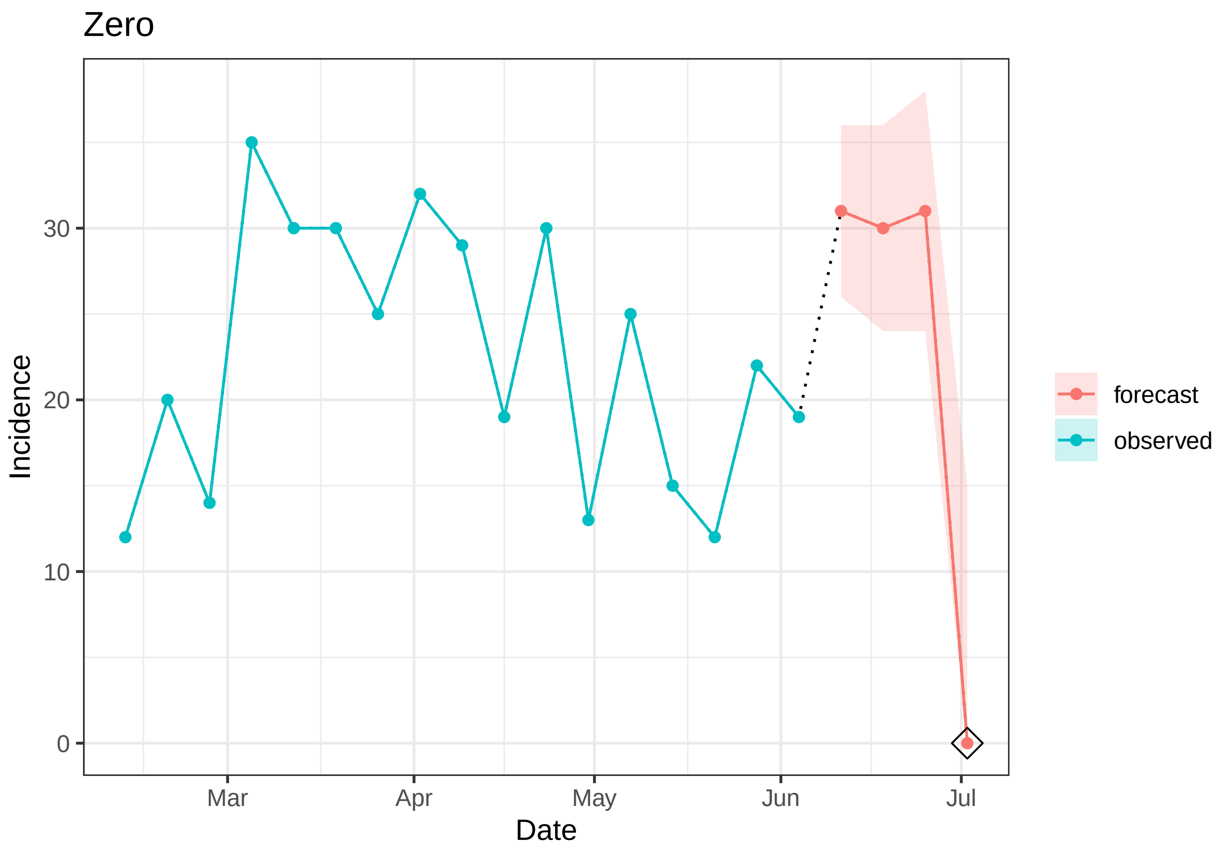

Depiction of a flag raised with the zero component. This component checks for the presence of any value equal to zero in the evaluated signal. If there are any zeros found, then the component will look in the seed to see if there are zeros anywhere else in the time series. If so, the component will consider the evaluated zero plausible and no flags will be raised. If not, the component will consider the evaluated zero implausible and a flag will be raised.
