# Supplementary material for: PLANES: Plausibility analysis of epidemiological signals
Source: PLoS One. 2025 Mar 28;20(3):e0320442. doi: 10.1371/journal.pone.0320442 (PMC11952232; doi:10.1371/journal.pone.0320442)
Supplement: Attachment — Submitted filename: 2025-01-31-plos-one-reviewer-response.pdf [file pone.0320442.s007.pdf]

Thank you for providing a thorough and constructive set of comments on our submission of *PLANES: Plausibility Analysis of Epidemiological Signals*. We have made major revisions to the manuscript that address the specific comments raised during review. Below we respond to each point summarized in the feedback delivered by the editorial team. The original comments are formatted in bold. We are happy to respond with additional details as needed.

**1. Broader Applicability:**

- **While the focus is on respiratory diseases, additional discussion is needed on how PLANES could handle other epidemiological data types, such as vector-borne diseases or sparse datasets.**
- **Provide insights into adapting components for diverse surveillance contexts.**

In the original submission, we very briefly mentioned our usage of PLANES to explore select non-respiratory signals. We have expanded that discussion to clearly articulate our expectation that the algorithm will handle other non-respiratory signals reported and/or forecasted at either daily, weekly, or monthly cadence. We continue to acknowledge that, as with implementation for respiratory signals, future usage with non-respiratory signals may identify additional areas for improvement. We also have more fully explained how PLANES behaves with sparse data (see separate response below).

Our goal in adding more detail regarding handling of non-respiratory and sparse datasets was to communicate that PLANES was designed to be disease agnostic and can generally be used in plausibility analysis of observed and forecasted signals in diverse surveillance contexts.

**2. Handling Sparse or Poor-Quality Data:**

- **Clarify the method's performance when historical data is sparse or unreliable.**
- **Discuss strategies within PLANES for dealing with poor-quality or incomplete data.**

We acknowledge that it is important to clarify how PLANES handles sparse or poor-quality inputs. We have added a paragraph to the Discussion that goes into depth regarding the implications of having incomplete data when seeding plausibility characteristics. We explain that PLANES, as currently implemented, will notify users if input data contains missing values. However, PLANES does not adjust or impute data automatically. In the manuscript, we now suggest that users may consider upstream data preparation if data are anticipated to be sparse or poor-quality for some or all the surveillance locations.

**3. Flexibility and Weight Adjustments:**

- **Expand on the impact of adjustable weights and component exclusion on PLANES scores.**
- **Include case studies or simulations to illustrate performance variations under different parameter settings.**

The manuscript now includes new analyses of the impacts of weighting and/or excluding PLANES components. For these analyses, we simulated weighting schemes and used all possible combinations of components to score the demonstration data (2022-23 FluSight forecasts). We include detailed information in the Methods and Results section, along with our perspective on what these results practically suggest in the Discussion section. There are two new figures that summarize the analyses in the supplemental information.

**4. Component Selection and Thresholds:**

- **Explain the criteria used to select PLANES components and define thresholds for plausibility classification.**
- **Discuss plans for adding new components in the future.**

In describing the modularity of the PLANES implementation in the Discussion section, the manuscript now includes details on how our team arrived at the initial set of plausibility components. Additionally, we have clarified that the API is modular and can accommodate new components in the future. While there are no components actively being developed at this time, new heuristics could be added to the scoring system without changing the usability of the tool.

**5. Automation and Scalability:**

- **Reflect on the trade-off between manual review and scalability for large-scale surveillance.**
- **Consider adding a table summarizing the method's limitations and possible extensions.**

The manuscript continues to emphasize that ideally users should implement PLANES to guide rather than replace human review. However, in the Discussion section we now highlight possible challenges with large-scale surveillance and forecast review efforts. We also provide an example and suggest that it is possible to build automation systems around the rplanes API if users deem it necessary to bypass manual review.

Thanks so much for this suggestion to add the table summarize methodological limitations. We now include a table that summarizes several limitations, along with their

current mitigations and future enhancements. The table is available as supporting information.

**6. Practical Applications:**

- **Provide concrete examples, simulations, or real cases demonstrating PLANES' ability to enhance ensemble predictions in surveillance consortia.**

We have included more discussion around potential use-cases for PLANES to optimize ensembles. The discussion section expands on how PLANES could be practically applied as an ensemble weighting scheme. We felt that it was also important to add citations to related studies of ensemble weighting and optimization strategies for context.

**7. Ethical and Transparency Concerns:**

- **Address ethical concerns related to filtering signals, ensuring transparency, and preventing misuse of the method.**
- **Propose guidelines for responsible implementation.**

Thank you for highlighting the need to address ethical and transparency concerns. We agree this is an important consideration. To address these topics, we have included a paragraph in the Discussion section that articulates our recommendations for responsible implementation, particularly when it comes to reviewing and censoring operational forecasts.

**8. Global Applicability and Equity:**

- **Discuss how PLANES can be adapted to low- and middle-income countries or regions with limited data infrastructure.**

With this feedback in mind, we have added a paragraph to the Discussion that characterizes applications for PLANES in circumstances where data infrastructure may be limited. We detail the implications for monitoring surveillance data quality in this context, particularly in low- and middle-income countries. We also highlight how the relative computational efficiency and accessibility of the rplanes package can be beneficial in these situations.
